# MiR-135a-5p suppresses breast cancer cell proliferation, migration, and invasion by regulating BAG3

**DOI:** 10.1016/j.clinsp.2022.100115

**Published:** 2022-10-10

**Authors:** Hongxu Zhang, Minghui Wang, Zhiqiang Lang, Haiwang Liu, Jianping Liu, Lihui Ma

**Affiliations:** a Departments of Breast Surgery, Affiliated Hospital of Chengde Medical University, Hebei, China; b Medical Research Center, Xi'an No.3 Hospital, Shaanxi, China.; c Department of Pathology, Affiliated Hospital of Chengde Medical University, Hebei, China

**Keywords:** Breast cancer, Mir-135a-5p, Bag3, Proliferation, Migration

## Abstract

**Background:**

MicroRNAs (miRNAs) are involved in the progression of diverse human cancers. This work aimed to delve into how microRNA-135a-5p (miR-135a-5p) affects the biological behaviors of Breast Cancer (BC) cells.

**Methods:**

Gene Expression Omnibus (GEO) datasets were used to analyze the expression differences of miR-135a-5p in cancer tissues of BC patients. Quantitative real-time PCR and western blot were conducted to detect miR-135a-5p and Bcl-2 Associated Athanogene (BAG3) expression levels in BC tissues and cells, respectively. The proliferation, migration, invasion, and cell cycle of BC cells were detected by cell counting kit-8 assay, BrdU assay, wound healing assay, transwell assay, and flow cytometry. The targeted relationship between miR-135a-5p and BAG3 mRNA 3′UTR predicted by bioinformatics was further testified by a dual-luciferase reporter gene assay. Pearson's correlation analysis was adopted to analyze the correlation between miR-135a-5p expression and BAG3 expression. The downstream pathways of BAG3 were analyzed by the LinkedOmics database.

**Results:**

MiR-135a-5p was significantly down-regulated and BAG3 expression was significantly raised in BC tissues. MiR-135a-5p overexpression repressed the viability, migration and invasion of BC cells, and blocked cell cycle progression in G0/G1 phase while inhibiting miR-135a-5p worked oppositely. BAG3 was verified as a target of miR-135a-5p. Overexpression of BAG3 reversed the impacts of miR-135a-5p on the malignant biological behaviors of BC cells. The high expression of BAG3 was associated with the activation of the cell cycle, mTOR and TGF-β signaling pathways.

**Conclusion:**

MiR-135a-5p regulates BAG3 to repress the growth, migration, invasion, and cell cycle progression of BC cells.

## Highlights

• MiR-135a-5p was significantly down-regulated and BAG3 expression was significantly raised in BC tissues.
• MiR-135a-5p overexpression inhibited the viability, migration and aggressiveness of BC cells, and blocked cell cycle progression in G0/G1 phase.
• Over-expression of BAG3 reversed the impacts of miR-135a-5p on BC cells.

## Introduction

Breast Cancer (BC) is one of the most common malignancies in females.^1^, ^2^, ^3^ The targeted therapy, an emerging treatment in addition to surgery, radiotherapy and chemotherapy, has the advantages of strong specificity, significant efficacy, low toxicity and side effects, improving the prognosis of the patients.^4^ With the deepening of molecular biology research, breakthrough progress has been made in exploring the potential targets of BC.

MicroRNA (miRNAs) are non-coding small RNA transcripts consisting of 17–25 *nt* that complement the 3′UTR of a specific target mRNA, resulting in mRNA degradation and translation inhibition.^5^ MiRNAs can function as tumor promoters or suppressors, and they are considered as promising therapeutic targets for cancers.^6^ The dysregulation of miRNAs is closely associated with the malignant biological behaviors of BC cells such as growth, apoptosis, invasion and angiogenesis.^7^ MiR-135a-5p partakes in the progression of a variety of malignancies. For example, miR-135a-5p overexpression restrains the growth and invasion of SW620 cells, and induces apoptosis;^8^ miR-135a-5p is significantly inhibited in glioma tissues, and miR-135a-5p overexpression reduces the growth of glioma xenografts in mouse.^9^ However, its biological function and mechanism in BC deserve further investigation.

Bcl-2 Associated Athanogene 3 (BAG3) is overexpressed in several tumors, and it is related to the growth, invasion, metastasis, drug resistance of tumor cells, and prognosis of the patients, making BAG3 a potential therapeutic target.^10^ Reportedly, BAG3 is highly expressed in the tumor tissues of patients with BC, and its high expression indicates a poor prognosis of the patients; knocking down BAG3 significantly suppresses the growth and migration of BC cells.^11^

This study is an in vitro study. We investigated the expression characteristics, biological function and underlying mechanism of miR-135a-5p. In this work, bioinformatics analysis suggested that miR-135a-5p was lowly expressed in BC tissues, and BAG3 may be a target of miR-135a-5p.

## Materials and methods

### Tissue sample collection

49 BC patients admitted to Xi'an No.3 Hospital from July 2017 to November 2021 were selected, and the surgically removed cancer tissues and corresponding para-cancer tissues were collected. The samples were stored in liquid nitrogen within 30 min after collection. All the individuals were not treated with radiotherapy, chemotherapy, and other related anti-cancer treatments before surgery. This work, with signed informed consent forms, was approved by the ethics committee of the hospital (2017YHDYY012) and all experiments were conducted according to the Declaration of Helsinki.

### Cell culture

BC cell lines (MCF-7, MDA-MB-231, MDA-MB-468 and DU4475) and HEK-293 cells were obtained from American Type Culture Collection (Manassas, VA, USA). Immortalized human mammary epithelial cells MCF-12A were obtained from BeNa Culture Collection (Beijing, China). These cells were cultured in Roswell Park Memorial Institute Medium 1640 (RPMI-1640, Gibco, Carlsbad, CA, USA) containing 10% fetal bovine serum (FBS; HyClone, Logan, UT, USA), 100 U/ml penicillin (Invitrogen, Carlsbad, CA, USA) and 100 μg/mL streptomycin (Gibco, Waltham, MA, USA) at 37 °C in 5% CO_2_. Once reaching about 90% confluence, the cells were trypsinized with 0.25% trypsin (Roche, Basel, Switzerland) for passage.

### Cell transfection

MDA-MB-231 and MCF-7 cells in the logarithmic growth phase were trypsinized with trypsin, inoculated into six-well plates at 1 × 10^6^ cells/well, and cultured overnight. Subsequently, the transfection was performed. MiRNA mimics control (miR-NC), miR-135a-5p mimics (miR mimics), miR-135a-5p inhibitors (miR inhibitors) and its Negative Control (inh-NC), empty plasmid (NC), BAG3 overexpression plasmid (BAG3), small interfering RNA targeting BAG3 (si-BAG3) and control siRNA Negative Control (si-NC) were transfected into MDA-MB-231 and MCF-7 cells according to the manufacturer's instructions of Lipofectamine™ 2000 kit (Invitrogen, Carlsbad, CA, USA). The above oligonucleotides and plasmids were available from RiboBio Co, Ltd. (Guangzhou, China). 48h later, the transfection efficiency was validated by quantitative Real-time PCR (qRT-PCR), and then the cells were collected for further analysis.

### qRT-PCR

The total RNA of tissue samples and cells was extracted using TRIzol reagent (Invitrogen, Shanghai, China) and reversely transcribed by a reverse transcription kit (Qiagen, Hilden, Germany) to generate cDNA. Reverse transcription conditions: 16 °C, 30 min; 42°, 30 min; 75 °C, 15 min. With cDNA as the template, qRT-PCR was then performed with a SYBR Premix-EX-TAQ (Takara, Tokyo, Japan) on an ABI7300 system (Thermo Fisher Scientific, Waltham, MA, USA). GAPDH or U6 was used as an internal control for mRNA or miRNA respectively. Primer sequences: miR-135a-5p: 5′-TTGGTCTTGTTTCCCGGTCC-3′ (forward), 5′-TCACAGCTCCACAGGCTAAC-3′ (reverse); BAG3: 5′-CAACAGCCGCACCACTAC-3′(forward), 5′-CATTGGCAGAGGATGGAGTC-3′ (reverse); U6: 5′-CTAGCTTCGGCAGCACA-3′(forward), 5′-AACGCTTCACGAATTTGCGT-3′ (reverse); GAPDH: 5′-ACAGTCAGCCGCATCTTCTT-3′ (forward), 5′-AAATGAGCCCCAGCCTTCTC-3′ (reverse). The PCR amplification conditions: 95 °C, 2 min; 95 °C, 15 s; 72 °C, 35 s for 35 cycles. The Cycle Threshold (CT) values were recorded, and the relative expression of miR-135a-5p and BAG3 mRNA were calculated by 2^−ΔΔCt^ method.

### Cell counting kit-8 (CCK-8) assay

After transfection, MDA-MB-231 and MCF-7 cells were inoculated in 96-well plates with 2000 cells per well and cultured for 24 h. According to the manufacturer's instruction, 10 μL of CCK-8 reagents (Med Chem Express, Monmouth Junction, NJ, USA) were added to each well at the 24th h, 48th h and 72nd h, and then the cells were incubated for an additional 1 h. The absorbance (OD) values of each well at 450 nm wavelength were detected at each indicated time point by a microplate reader (Bio-Rad, Hercules, CA, USA).

### BrdU assay

MDA-MB-231 and MCF-7 cells were inoculated into 24-well plates (with a cover glass inside) at 2.5 × 10^5^ cells/well. 24 h later, BrdU solution (Beyotime Biotechnology, Shanghai, China) was loaded, followed by incubation for 4 h. Subsequently, the cells were fixed with 4% paraformaldehyde for 30 min. Next, the cells were stained with 100 μL of 1 × Apollo for 30 min. Next, l g/mL DAPI staining solution was dripped into each well for incubation in darkness for 20 min at ambient temperature. The cells were washed with Phosphate Buffer Saline (PBS), and the total number of cells and the number of BrdU-positive cells in the randomly chosen 10 high-power fields were counted under a microscope. The positive rate of BrdU staining was then calculated.

### Transwell assay

Transfected MDA-MB-231 and MCF-7 cells were trypsinized with trypsin and followingly centrifuged at 500 × g for 5 min to collect the cells. After the cell density was adjusted with a serum-free medium, a cell suspension of 1 × 10^5^ cells/mL was prepared. In the migration assay, 100 μL of cell suspension was added into the upper compartment of the transwell chamber (8 μm pore size), and 650 μL of medium with 10% FBS was added into the lower compartment, and the cells were cultured in 5% CO_2_ at 37 °C for 24 h. Next, the chambers were taken out, and the cells on the upper surface of the microporous membrane were wiped off with a cotton swab. The chamber was carefully rinsed with PBS two times, and the cells on the lower side of the microporous membrane were fixed with 4% paraformaldehyde for 15 min, and then stained with 0.1% crystal violet for 20 min. Next, the chamber was immersed in PBS, and air-dried. The cells were observed under a microscope, and the number of cells was counted. For the invasion assay, the microporous membrane was covered with a layer of Matrigel, and the remaining steps were the same as those in the migration assay.

### Flow cytometry

The transfected cells were inoculated into a 6-well plate (1 × 10^5^ cells/well) and cultured overnight, and then the cells were trypsinized with trypsin, washed with Phosphate Buffer Saline (PBS), harvested, and re-suspended in 300 μL of binding solution. Then the cells were mixed with and incubated with 10 μL of Annexin V-FITC staining solution (BD Bioscience, San Diego, CA, USA) for 15 min in the darkness at ambient temperature. Then, 5 μL of Propidium Iodide (PI) staining solution (BD Bioscience, San Diego, CA, USA) was added for staining the cells for 5 min in darkness. Finally, the cells were washed with PBS, and the apoptosis of the transfected cells was detected by a flow cytometer.

### Dual-luciferase gene reporter assay

The binding site between miR-135a-5p and BAG3 3′UTR was predicted by bio information. A fragment with the binding site for miR-135a-5p of BAG3 mRNA 3′UTR was amplified by PCR and followingly inserted into a pMIR-REPORT luciferase vector (Ambion, Frederick, MD, USA) to construct a BAG3 Wild-Type plasmid (WT-BAG3). Then, partial nucleotides at the binding site were mutated by Gene Mapping Techniques to construct a mutated reporter plasmid (MUT-BAG3). HEK-293 cells were co-transfected with the reporter plasmids and miR-mimics (or miR-inhibitors) or miR-NC (or inh-NC) to verify the binding relationship between miR-135a-5p and BAG3 mRNA 3′UTR. 36 h later, according to the manufacturer's instructions of the luciferase activity detection kit (Ambion, Frederick, MD, USA), the relative luciferase activity of the cells in each group was detected, expressed as the ratio of the firefly luciferase activity to the Renilla luciferase activity.

### Western blot

The transfected BC cells were harvested, and the total protein was extracted by ice-cold RIPA lysis buffer (Pierce, Rockford, IL, USA). The protein was separated by sodium dodecyl sulfate-polyacrylamide gel electrophoresis and followingly transferred to polyvinylidene fluoride membranes, which were blocked with 5% skimmed milk for 1 h at room temperature, and then the membranes were incubated with primary recombinant anti-BAG3 antibody (ab92309, 1:1000, Abcam, Shanghai, China) for 2 h at room temperature. The membranes were then washed and then incubated with goat-anti-rabbit IgG H&L (ab216773, 1:5000, Abcam, Shanghai, China) for 1.5 h at room temperature, followed by washing again. Finally, the protein bands were developed by the enhanced chemiluminescence detection reagent (Beyotime, Shanghai, China), and analyzed by a gel imaging system. The ratio of the density of target protein to the density of the internal reference was taken as the relative expression of the target protein.

### Statistical analysis

SPSS 22.0 software was adopted for data analysis. The data conformed to normal distribution were expressed as “*x* ± s*”*. The student's *t*-test was executed for comparisons between two groups, and a one-way analysis of variance was conducted for the comparisons among multiple groups. The correlation analysis was performed with Pearson's correlation analysis; *p* < 0.05 is indicative of a statistically significant difference.

## Results

### Expression characteristics of miR-135a-5p in BC

**Fig. 1:**
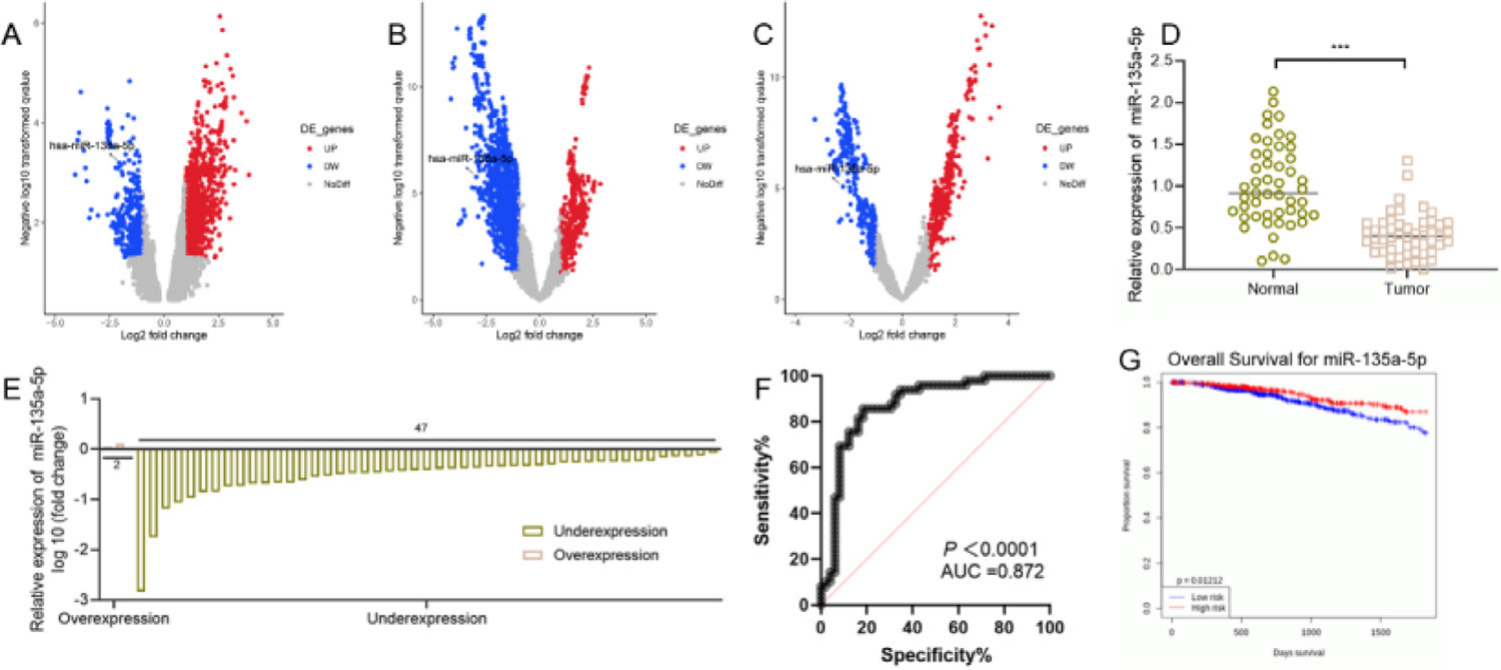
**Expression characteristics of miR-135a-5p in tissues of BC patients and BC cells.** (A‒C) The microarray datasets GSE80038, GSE45666 and GSE38167 were downloaded from the GEO database to analyze the expression differences of miRNA, and the volcano map was used to display the differentially expressed miRNAs in the BC samples vs. normal tissue samples. (D, E) The expression of miR-135a-5p in the cancer tissues and normal tissues of 49 BC patients was detected by qRT-PCR. (F) ROC curve analysis showed the diagnostic accuracy of miR-135a-5p. (G) OncomiR database was used to analyze the relationship between the expression of miR-135a-5p and the overall survival of patients with BC. *p < 0.05, ** *p* < 0.01, and *** *p* < 0.001.

Gene Expression Omnibus (GEO) database showed that 339 miRNAs were down-regulated, and 1492 miRNAs were up-regulated in dataset GSE80038; in dataset GSE45666, 1285 miRNAs were down-regulated and 394 miRNAs were up-regulated; in dataset GSE38167, 343 miRNAs were down-regulated and 378 miRNAs were up-regulated, of which, miR-135a-5p was markedly inhibited in BC tissues in all of the three datasets (Fig. 1A-C). qRT-PCR showed that miR-135a-5p expression in cancer tissues of BC patients was down-regulated compared with that in the normal group (Fig. 1D). Moreover, miR-135a-5p expression was down-regulated in BC sample of 95.9% cases (47 of 49) (Fig. 1E). Receiver Operating Characteristic (ROC) curve analysis suggested that miR-135a-5p could be considered as a discriminative biomarker for BC with 85.71% sensitivity and 81.63% specificity in the optimal cutoff value = 0.6069 (AUC = 0.872; *p* < 0.001), (Fig. 1F). In addition, low expression of miR-135a-5p was associated with lymph node metastasis and higher TNM stage of the patients (*p* < 0.05) (Table 1). In addition, the OncomiR database showed that low miR-135a-5p was significantly associated with short overall survival in patients (Fig. 1G).

**Table 1:** The correlation between clinicopathological features and miR-135a-5p expression in BC patients.

Pathological parameters	Numbers (*n* = 49)	MiR-135a-5p expression		p*-*value
		High (*n* = 25)	Low (*n* = 24)	
Age (years)				0.296
< 35	20	12	8	
≥ 35	29	13	16	
Lymphatic metastasis				0.013^a^
Negative	21	15	6	
Positive	28	10	18	
ER status				0.889
Negative	25	13	12	
Positive	24	12	12	
PR stage				0.054
Negative	16	5	11	
Positive	33	20	13	
TNM stage				0.004^a^
Ⅰ + Ⅱ	18	14	4	
Ⅲ + Ⅳ	31	11	20	
Tumor size (cm)				0.469
≥ 2	26	12	14	
< 2	23	13	10	

ER, Estrogen Receptor; PR, Progesterone Receptor; TNM, Tumor, Node, Metastasis staging classification.

a Means that the statistical difference less than  0.05.

### The biological function of miR-135a-5p in BC

**Fig. 2:**
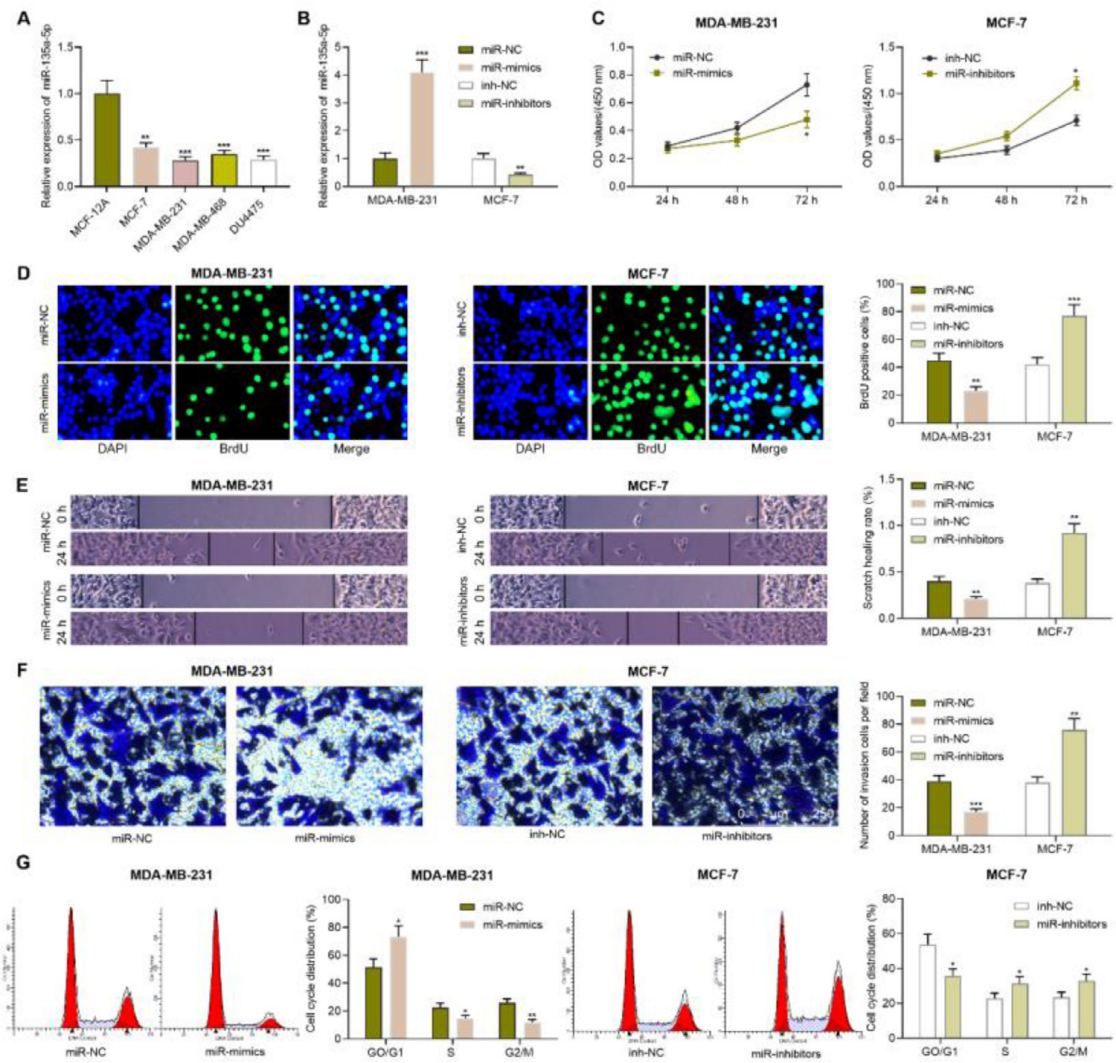
**Impacts of miR-135a-5p on the proliferation, migration, invasion and cell cycle progression of BC cell.** (A) The expression of miR-135a-5p in immortalized mammary epithelial cells and BC cell lines was detected by qRT-PCR. (B) miR-NC, miR-mimics and inh-NC, and miR-inhibitors were transfected into MDA-MB-231 cells and MCF-7 cells, respectively, and the transfection efficiency was detected by qRT-PCR. (C) After transfection, MDA-MB-231 and MCF-7 cells were tested for the viability by CCK-8 assay. (D) After transfection, the proliferation of MDA-MB-231 and MCF-7 cells was detected by BrdU method. (E, F) Changes in the migration and invasion abilities of MDA-MB-231 and MCF-7 cells after transfection were detected by wound healing assay and Transwell assay. (G) Changes in the cell cycle distribution of MDA-MB-231 and MCF-7 cells were detected by flow cytometry after transfection. * *p* < 0.05, ** *p* < 0.01, and *** *p* < 0.001.

Compared with MCF-12A, miR-135a-5p expression in the BC cell lines (MCF-7, MDA-MB-231, MDA-MB-468, DU4475) was significantly down-regulated (Fig. 2A). Among the five BC cell lines, miR-135a-5p expression in MDA-MB-231 cells was the lowest, while that of MCF-7 cells was the highest. Therefore, we transfected miR-mimics (or miR-NC) and miR-inhibitors (or NC-inhibitors) into MDA-MB-231 cells and MCF-7 cells, respectively, to construct the cell models with miR-135a-5p overexpression or inhibition, with qRT-PCR testifying it a success (Fig. 2B). CCK-8 assay showed that as against the control, miR-135a-5p overexpression suppressed the viability of MDA-MB-231 cells while inhibiting miR-135a-5p promoted the cell viability of MCF-7 (Fig. 2C). Next, BrdU assay, transwell assay, and flow cytometry showed that miR-135a-5p overexpression restrained the growth, migration, and invasion of BC cells and arrested the cell cycle progression at G0/G1 phase; inhibiting miR-135a-5p had the opposite effect (Fig. 2D-G).

### BAG3 is a downstream target of miR-135a-5p

**Fig. 3:**
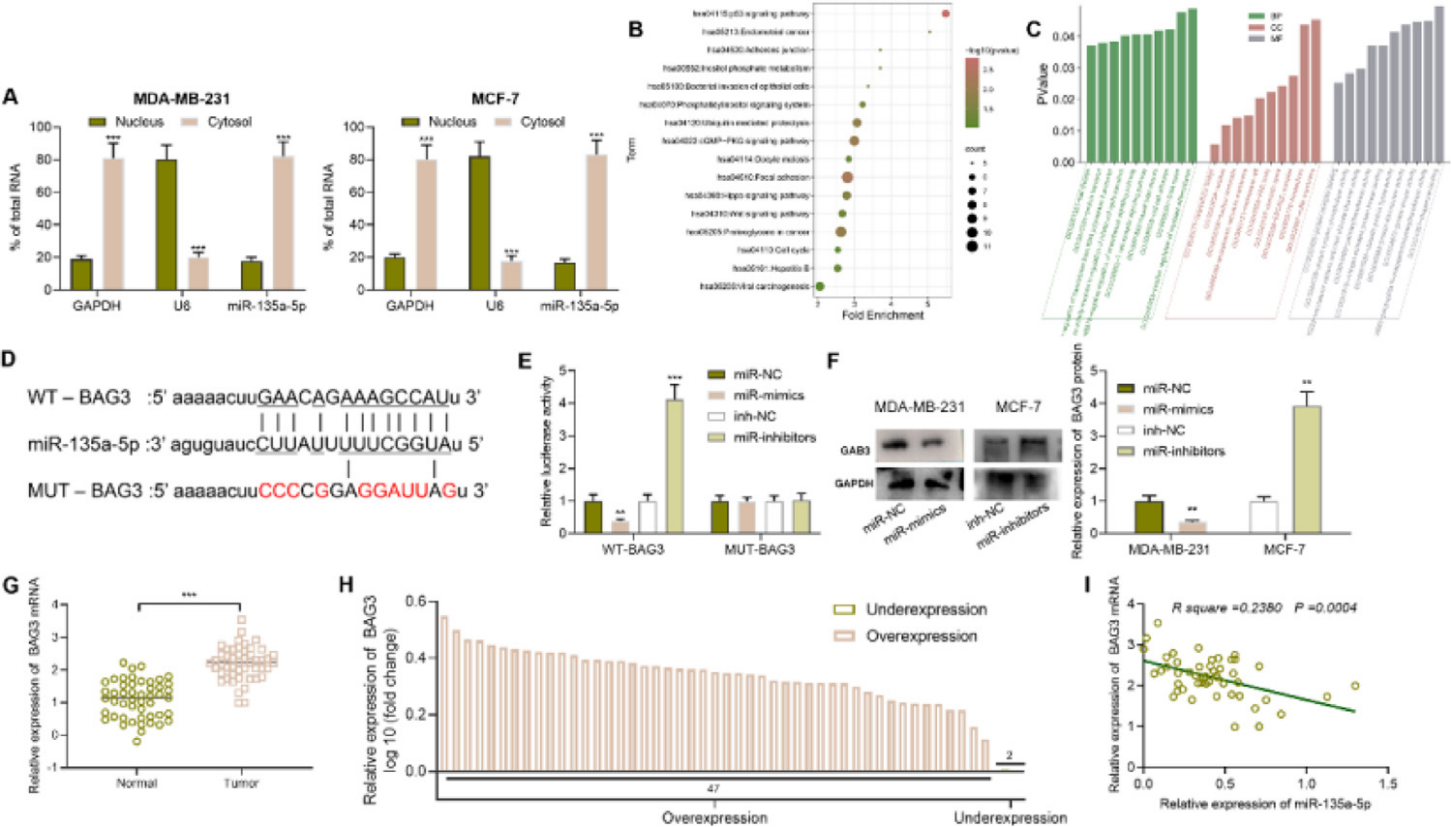
**BAG3 is a direct downstream target of miR-135a-5p.** (A) The subcellular localization of miR-135a-5p was detected by qRT-PCR in BC cells. (B) KEGG pathway analysis of the downstream target genes of miR-135a-5p. (C) GO enrichment analysis of the downstream target genes of miR-135a-5p. (D) The binding sequence of BAG3 mRNA 3′ UTR wild-type and mutant sequences with miR-135a-5p. (E) The effect of the miR-135a-5p overexpression on the luciferase activities of WT-BAG3 and MUT-BAG3 was verified by dual luciferase reporter gene assay. (F) Western blot was used to detect the effect of the overexpression or inhibition of miR-135a-5p on the expression of BAG3 protein. (G, H) The expression of BAG3 mRNA in the tumor tissues and normal tissues of 49 BC patients was detected by qRT-PCR. (I) Pearson's correlation analysis was used to analyze the correlation between BAG3 mRNA expression and miR-135a-5p expression in BC tissues. *p < 0.05, ** *p* < 0.01, and *** *p* < 0.001.

**Fig. 4:**
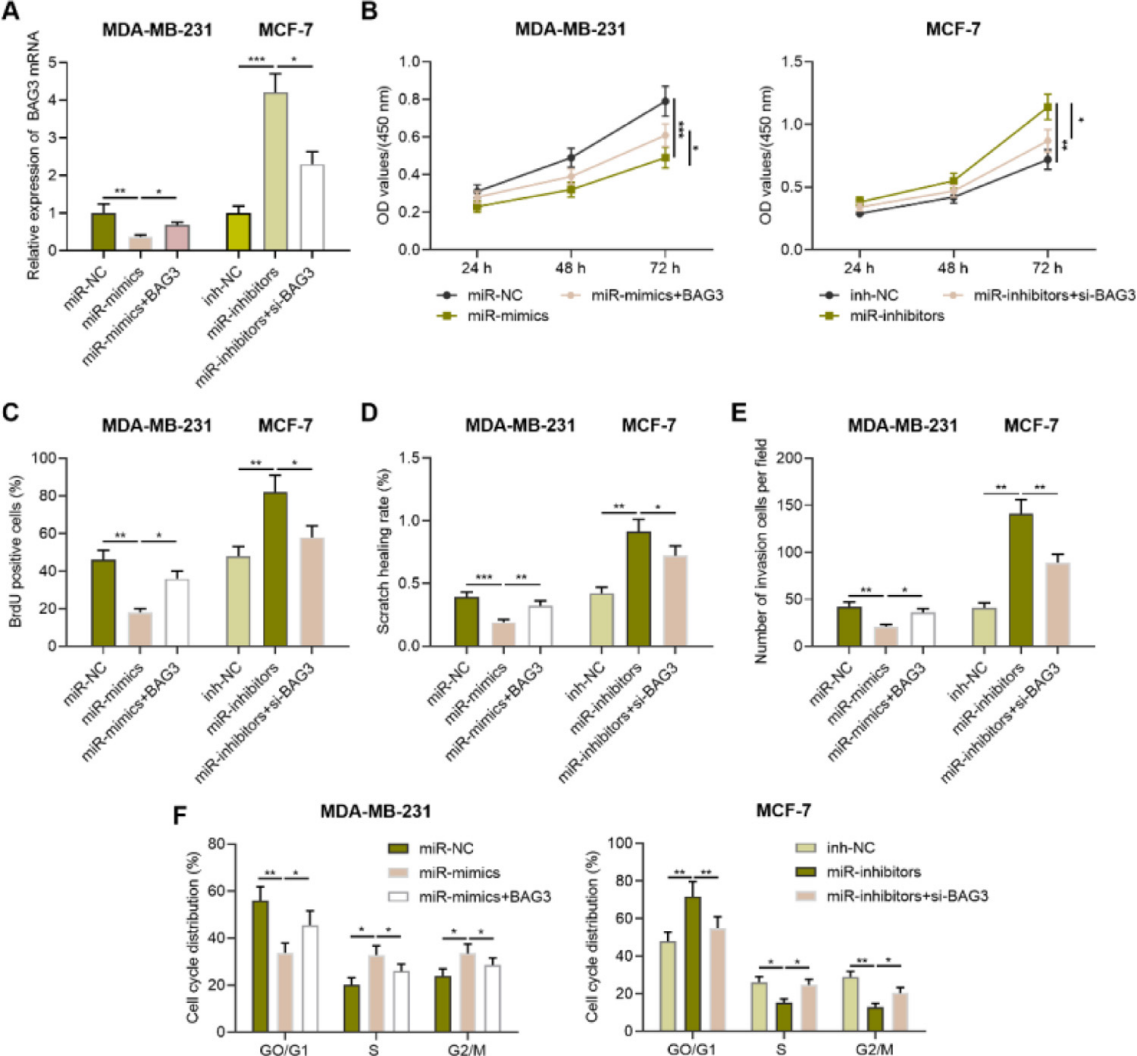
**BAG3 reverses the effects of miR-135a-5p on the proliferation, migration, invasion, and cycle of BC cell.** (A) miR-NC, miR-mimics, miR mimics+BAG3 and inh-NC, miR-inhibitors, and miR-miR inhibitors+si-BAG3 were co-transfected into MDA-MB-231 and MCF-7 cells, respectively, and the transfection efficiency was detected by qRT-PCR. (B) After transfection, MDA-MB-231 and MCF-7 cells were tested for the viability by CCK-8 assay. (C) After transfection, the proliferation of MDA-MB-231 and MCF-7 cells was detected by BrdU method. (D, E) Changes of the migration and invasion abilities of MDA-MB-231 and MCF-7 cells after transfection were detected by wound healing assay and Transwell assay. (F) Changes of cell cycle of MDA-MB-231 and MCF-7 cells after transfection were detected by flow cytometry. *p < 0.05, ** *p* < 0.01, and *** *p* < 0.001.

We then identified the subcellular localization of miR-135a-5p in MDA-MB-231 and MCF-7 cells by qRT-PCR and found that miR-135a-5p was mainly present in the cytoplasm of BC cells, suggesting it may regulate gene expression at the post-transcriptional level (Fig. 3A). To expound the mechanism of miR-135a-5p, StarBase database was searched, and the downstream genes of miR-135a-5p were analyzed by DAVID database. Kyoto Encyclopedia of Genes and Genomes (KEGG) enrichment analysis showed that the above target genes were involved in the p53, Hippo, and Wnt signaling pathways, all of which were associated with cancer biology (Fig. 3B). Gene Ontology (GO) analysis showed that the above target genes were significantly enriched in biological processes such as cell division and protein transport; they were also enriched in cellular components including intercellular bridge and nucleus, etc.; and in molecular functions including protein complex binding and protein tyrosine phosphatase activity (Fig. 3C). Among the candidate targets, BAG3, which is closely associated with BC progression, attracted the authors’ attention, and the binding sequence was shown in Fig. 3D. Subsequently, dual luciferase reporter gene assay showed that up-regulating miR-135a-5p remarkably suppressed the luciferase activity of WT-BAG3 reporter and inhibiting miR-135a-5p significantly increased the activity of WT-BAG3 reporter, while that of MUT-BAG3 group was not significantly impacted by miR-135a-5p (Fig. 3E). Western blot showed that miR-135a-5p overexpression down-regulated BAG3 expression, while its inhibition functioned oppositely (Fig. 3F). Through qRT-PCR, we observed that BAG3 mRNA expression in BC tissues was increased compared with that in the control group (Fig. 3G), and BAG3 mRNA expression was increased in 95.9% cases (47 of 49 cases) (Fig. 4H), and BAG3 mRNA was negatively correlated with miR-135a-5p expression in BC samples (Fig. 3I).

### BAG3 counteracts the impact of miR-135a-5p on the biological behaviors of BC cells

To further explore the effects of miR-135a-5p and BAG3 on the growth, migration, invasion, and cell cycle progression of BC cells, miR-NC, miR mimics, miR mimics+BAG3 and inh-NC, miR inhibitors, and miR inhibitors+si-BAG3 were co-transfected into MDA-MB-231 and MCF-7 cells, respectively, with qRT-PCR assay showing it a success (Fig. 4A). CCK-8 assay, BrdU assay, wound healing assay, transwell assay and flow cytometry assay showed that miR-135a-5p overexpression significantly inhibited cell growth, migration, and invasion and blocked cell cycle progression in G0/G1 phase compared with the control group, and overexpressing BAG3 reversed these effects; miR-135a-5p inhibition remarkably promoted the malignant biological behaviors of BC cells, while silencing BAG3 counteracted these effects (Fig. 4B-F).

### BAG3 activates the cell cycle, mTOR, and TGF-β signaling pathway

**Fig. 5:**
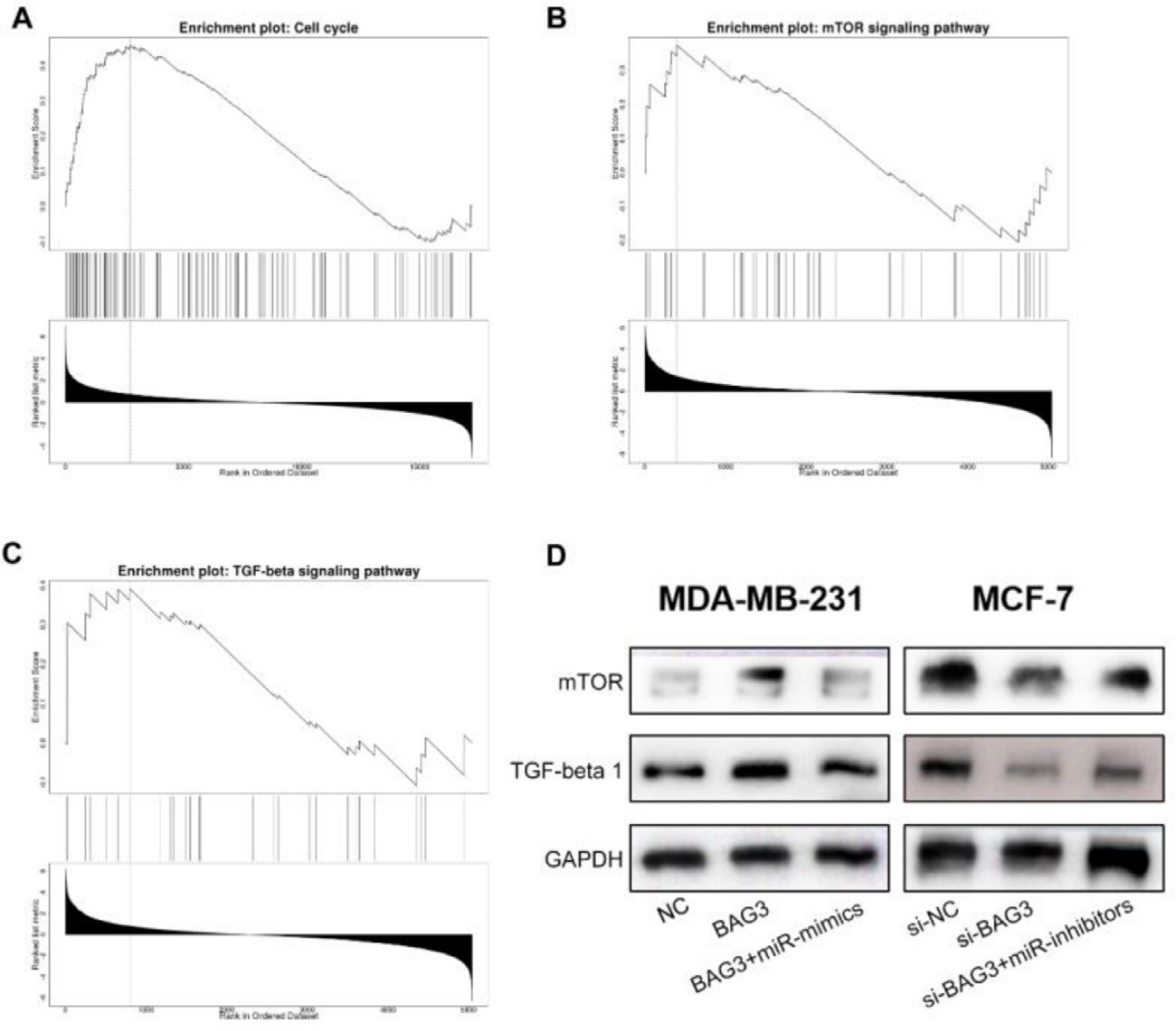
**BAG3 activates the cell cycle, mTOR and TGF-β signaling pathway.** (A‒C) GSEA plot showed that BAG3 expression was associated with cell cycle and mTOR pathway and TGF-β signaling pathway. (D) Western blot was performed to detect the expression of mTOR and TGF-β1 after transfection of BAG3, BAG3+miR-mimics and si-BAG3, BAG3+miR-inhibitors into MDA-MB-231 and MCF-7 cells, respectively.

To further clarify how miR-135a-5p/BAG3 axis regulates the biological behaviors of BC cells, we used Gene Set Enrichment Analysis (GSEA) to predict the signaling pathways possibly associated with BAG3. The results showed that the high expression of BAG3 was positively associated with the activation of the cell cycle, mTOR and TGF-β signaling pathways (Fig. 5A-C). Subsequently, Western blot was showed that overexpression of BAG3 up-regulated the expressions of mTOR and TGF-β1, and miR-135a-5p overexpression blocked these effects; knockdown of BAG3 down-regulated the expression of mTOR and TGF-β1, while inhibition of miR-135a-5p worked oppositely (Fig. 5D).

## Discussion

The tumorigenesis and progression of BC involve the activation of proto-oncogenes and the inactivation of tumor suppressors, followed by the dysregulation of signal pathways, and finally the change of phenotypes of cells.^12^^,^^13^ MiRNAs are conceptually a group of endogenous short non-coding RNA transcripts, which are involved in biological processes such as cell growth, apoptosis, and differentiation.^14^ MiR-135a-5p has different expression patterns in different cancers: high expression of miR-135a-5p in colon cancer tissues is associated with unfavorable clinicopathologic indexes of the patients; inhibiting miR-135a-5p suppresses the growth, migration, and invasion of SW620 cells.^8^ However, miR-135a-5p is under-expressed in head and neck squamous cell carcinoma, which is closely related to the poor prognosis of the patients; miR-135a-5p overexpression targets HOXA10, thereby inhibiting tumor cell growth and promoting the apoptosis.^15^ In nasopharyngeal carcinoma, miR-135a-5p expression is also reduced; miR-135a-5p is negatively regulated by lncRNA FOXD3-AS1, and inhibiting miR-135a-5p can significantly promote the viability and inhibit the apoptosis of nasopharyngeal carcinoma cells.^16^ Here we found that miR-135a-5p expression was reduced in BC tissues, which was closely associated with the poor prognosis of the patients. Additionally, miR-135a-5p overexpression inhibited the malignant biological behaviors of BC cells, while miR-135a-5p inhibition exerted the opposite effects. The present data suggest that miR-135a-5p may serve as a tumor suppressor in BC.

In eukaryotes, autophagy is a highly conservative catabolism process in which cells degrade intracellular substances, organelles, or proteins through lysosomes and recycle various cytoplasmic components to provide energy and substrates for biosynthesis.^17^^,^^18^ Moreover, autophagy is quietly vital in the maintenance of cell homeostasis, cell differentiation, growth and development, and stress response, and the dysregulation of autophagy is associated with the pathogenesis of a lot of human diseases.^17^^,^^18^ As the regulatory molecule of autophagy, BAG3 plays a vital role in maintaining cell homeostasis, which has attracted extensive attention in recent years, especially in cancer biology.^19^ One of the important functions of BAG3 is to degrade misfolded proteins or protein aggregates by regulating selective macroautophagy/autophagy under stress conditions, thereby maintaining protein homeostasis,^20^ and it is involved in a series of physiological and pathological processes including growth, apoptosis, adhesion, and viral replication.^21^ Some previous studies have shown that in some human malignancies, such as thyroid cancer,^22^ cervical cancer,^23^ ovarian cancer,^24^ BAG3 is abnormally expressed, and it can modulate the progression of the disease. For example, BAG3 is abnormally highly expressed in ovarian cancer tissues and cells, and the knock-down of BAG3 suppresses the viability of tumor cell, enhances the sensitivity of tumor cells to Olaparib, and synergistically kills ovarian cancer cells in vitro with Olaparib via modulating autophagy.^24^ In addition, there are some previous studies on BAG3 in BC. For example, in BC, BAG3 up-regulates the expression levels of Bcl-2 and Bcl-xL by targeting HSP70/Mcl-1 signaling pathway and suppresses the chemoresistance of tumor cells, indicating that BAG3 may be a potential target for treating BC.^25^ Additionally, high expression of BAG3 in triple-negative BC is associated with poor prognosis of patients, and BAG3 regulates PI3K/AKT and FAK/Src pathways to promote the proliferation, migration, and invasion of triple-negative BC cells.^11^ In the present work, we found that BAG3 was the downstream target of miR-135a-5p. miR-135a-5p specifically modulated BAG3 in BC cells, further regulating mTOR and TGF-β pathways. These data partly explain the mechanism of BAG3 dysregulation in BC tissues/cells.

In summary, miR-135a-5p expression is reduced in BC and its expression level is associated with the prognosis of patients. Additionally, miR-135a-5p negatively regulates BAG3 expression, inhibits mTOR and TGF-β pathways, and restrains the growth, migration, and invasion of BC cells. This study suggests that miR-135a-5p may be a potential biomarker and therapeutic target for the diagnosis and treatment of BC.

## Author's contribution

Conceived and designed the experiments: Zhiqiang Lang, Hongxu Zhang, Lihui Ma.

Performed the experiments: Zhiqiang Lang, Haiwang Liu.

Analyzed the data: Hongxu Zhang, Minghui Wang, Jianping Liu.

Wrote the paper: Hongxu Zhang, Minghui Wang, Zhiqiang Lang.

All authors read and approved the final manuscript.

## Funding information

This study was supported by the Science and Technology Support Program of Chengde (201801A032).

## Ethics statement

The present study was approved by the Ethics Review Board of Xi'an No.3 Hospital (Ethics committee number: 2017YHDYY012).

## Data availability statement

The data used to support the findings of this study are available from the corresponding author upon request.

## Declaration of Competing Interest

The authors declare no conflicts of interest.
